# Retroversion of the Pregnant Womb

**Published:** 1868-09-01

**Authors:** F. R. Mergler

**Affiliations:** Wheeling, Cook County, Ill.


### RETROVERSION OF THE PREGNANT WOMB.

BY F. R. MERGLER, M.D., WHEELING, COOK COUNTY, ILL.

PROF. ALLEN,—Having read with interest the paper of our medical brother, Dr. Bogue, published in the *Chicago Medical Journal* for July 15th, concerning that alarming accident of a pregnant woman, termed "*Retroversio Uteri*, I take the liberty to present you for your readers some lines on the same malady, together with a few cases, showing that this displacement can be successfully combated if we try to replace the womb *per rectum* with four fingers, instead of manipulating *per vaginam*, and that *abortus is avoided* in this manner. Lately the *colpeurynter* has been used for replacement of the womb. If we succeed by it, very well; but, if this elegant windbag should fail, too, it may be good to have another resource, which, in my experience, knoweth no failure.

In the year 1850, I had to meet my first case of *retroversio uteri* in a woman gone about three months. The principal trouble for which she applied, was an alarming *ischuria*. After due examination, *per vaginam*, I soon learned the real cause of her complaint, emptied the bladder by aid of the catheter, and then proceeded to restore the displaced uterus, manipulating (according to the teachings of books and professors) within the vaginal canal. But, as often as I pressed my finger against the posterior wall of the vagina, and upward against the impacted uterus, I failed constantly in every effort. Discouraged, I caused counsel to be procured, and although the venerable consiliarus did his best, no replacement was effected. He had been manipulating in the same manner as I did—*per vaginam.* A few days afterwards, as could be expected, *abortus took place.*

Shortly after this sad disappointment I happened to see Dr. F. Kiwisch von Rotterhaus' recent publication on female diseases. Naturally, with the highest interest and curiosity, I studied his chapter on "*Retroversio Uteri Gravidi.*" As soon as I had learned his advice for this accident, I felt at first not a little sorry, because I was now convinced that the above narrated unhappy success in my first case of *retroversio* would not have occurred, if I had sooner been acquainted with the method of Kiwisch. But I could not do any better than to press firmly into my memory his mode of operation, determined to try its merits as soon as occasion was given. I am unable to say whether Kiwisch is the real inventor of this *modus operandi*, but I had never before read any similar advice. It is chiefly this:

1. Empty the bladder with a *male* (not female) catheter. The cervix uteri in this accident being displaced forwards and upwards, presses firmly against the os pubis, and compresses, therefore, the neck of the bladder so strongly, that a real occlusion of it is effected. Hence, a well bent male catheter is more apt to enter the bladder by creeping around the *symphysis osseum pubis* upwards and forwards. A silver catheter may be preferable to an elastic one, and an index finger introduced into the vagina during the introduction of the catheter, should guard the blunt point of the catheter, and help to lead it around the symphysis, otherwise perforation of the neck of the bladder might be effected.

Having first emptied the over-extended bladder, you have removed one obstacle to the return of the womb to its normal place.

II. The next obstacle against the return of the womb in its place is that osseous projection from the sacral bone, known as the *promontorium pelvis.* The body of the womb, having been, by some mechanical cause, bent and forced back and downwards into the hollow of the sacrum, is held there fast and prevented from re-ascension to its former erect position by the promontory. To overcome this difficulty, manipulation within the vagina is many times insufficient. We are better able to lift the uterus out of its malposition, *if we succeed in bringing the operating fingers between the uterus and sacrum.* Therefore, let the patient take a position on her knees, bending the head and upper part of the body down as far as possible. Lubricate your operating hand very well, and insert the first two fingers of it into the rectum of the patient. You will now detect through the *anterior* wall of the rectum, a roundish, elastic body, projecting below the promontory into the sacral excavation. If your fingers are long enough, you may press them instantly against the uterine body. But you will seldom be able with one or two fingers to complete the operation. You must, therefore, cautiously introduce the other two fingers also, the thumb remaining outside. This dilatation of the sphincter ani with four fingers is the only painful act of the operation, and a somewhat rude proceeding. But is is pretty well practicable, and secures the success.

As soon as you have inserted your hand into the rectum, you should give to it such a turn, that its back looks towards the sacrum. Now, you may commence to press with the tips of your fingers gently against the womb. From one to three forward pushings with your fingers will lift the womb out of its sacral bed, and will induce it to glide readily along the lower side of the promontory forward and return (at most with a kind of joyous jump) back into its former erect position. A digital examination *per vaginam*, will instantly satisfy you that the uterus is now well located again, and the troubles ended.

This is a condensed delineation of the mode of replacement of the retroverted uterus, after Kiwisch, as I have afterwards practiced, and in *none* of the cases where I tried it, was I disappointed any more.

I will therefore, now, to illustrate the result of this mode of operation, report a few cases of *retroversio uteri* which I have met during my professional life, and which are, as I believe, highly encouraging.

Case I. (1858.) Mrs. V. of Lac Furic, Illinois; wife of a farmer; aged about forty years; of a very lax and feeble constitution; mother already of six children; pregnant again since three months. Sent for me, asking relief from a very troublesome ischuria. Examination proved very soon a case of retroversio of the pregnant uterus, existing about two days. Proceeded to make the replacement after Kiwisch's method, the first time operating *per rectum with the hand.* Operation succeeded satisfactorily.

Case II. Only fourteen days after this reposition of the womb, the same lady returned to my office, complaining as before. Made the replacement again in same manner successfully. No abortus afterwards occurred. No other troubles throughout the whole pregnancy were felt. At the termination of it, she was delivered of a full grown child by the aid of the forceps, which I had to use on account of absence of expulsory contraction of the womb. The child was dead. Mother recovered well, and gave birth afterwards to three other children. Had never again in any of her further pregnancies to complain of *retroversio.* But the two last of her children had each one to be taken from her with forceps. Mrs. V. having had her share now, since then she has enjoyed relatively good health up to the present time.

Case III. (1866.) Mrs. H., of Long Grove, Ill.; wife of a farm laborer; aged about thirty years; mother of several children; generally healthy and hard-working; pregnant for four months. She informed me that just four weeks before this she was attacked severely with ischuria. Her impotence to void her urine, turned after a few days of great suffering, to the other extreme. The urine flowing again could not be retained at all any more. Constantly, a blood-colored urine was dropping away from her, and wherever she was going, sitting or standing, the floor below was soiled with the water. This state of things was yet present, and I was not a little surprised that things had so long their way without medical interference.

The external examination of the poor sufferer's abdomen showed the bladder extended to a tremendous volume, so that it could be easily mistaken for the womb itself in highest pregnancy.

The examination *per vaginam* elucidated unmistakably another case of *retroversio uteri gravidi*. After the necessary explanations, I introduced first the male catheter, and abstracted not less than nearly three-quarters of a pail full of bloody urine of the most penetrant bad odor. After this, the abdomen came instantly down to a shape more adequate to a four months pregnancy. Then I made the reposition of the womb *per anum* with the hand (four fingers), as before described, and had the satisfaction to restore the poor woman in a short time to a normal state again. Re-examination *per vaginam* proved it instantly.

Mrs. H. from this time had no untoward symptom any further. In due and regular time (after five months more), she was delivered of a full grown child, which is alive and well.

Case IV. (1868). The same lady called at my office again about the 4th of July. Was anew pregnant since three months, and for the last two days troubled as once before with inability to void her urine. Examination proved that we had to combat another retroversion of the womb. This I did successfully as in the cases before, and Mrs. H. expects again a safe delivery when her time will come. At least, no abortus has yet occurred.

This is my experience in the line of *retroversio uteri* during a practice of twenty-eight years. If any one of our brethren, in case of emergency, after other methods and even the colpeurynter have failed—would try the above described method of *replacement per anum with the hand*, I feel confident that he will not, as I did, *experience disappointment*. The dilatation of the sphincter ani with the four fingers may be with many a serious objection, but having been compelled, as I was once, to resort to it, it will be found not half so dangerous as expected. Notwithstanding, I wish heartily that this rough proceeding may be spared to our patients, if there is any possibility.

